# RPGR: Its role in photoreceptor physiology, human disease, and future therapies

**DOI:** 10.1016/j.exer.2015.06.007

**Published:** 2015-09

**Authors:** Roly D. Megaw, Dinesh C. Soares, Alan F. Wright

**Affiliations:** a Scottish Centre for Regenerative Medicine, University of Edinburgh, 5 Little France Drive, Edinburgh EH16 4UU, United Kingdom; b Medical Research Council Human Genetics Unit, Institute of Genetics and Molecular Medicine, University of Edinburgh, Edinburgh EH4 2XU, United Kingdom

**Keywords:** Ciliopathy, Retinitis pigmentosa, RPGR, Human disease, Animal model, Stem cell, Gene therapy

## Abstract

Mammalian photoreceptors contain specialised connecting cilia that connect the inner (IS) to the outer segments (OS). Dysfunction of the connecting cilia due to mutations in ciliary proteins are a common cause of the inherited retinal dystrophy retinitis pigmentosa (RP). Mutations affecting the Retinitis Pigmentosa GTPase Regulator (RPGR) protein is one such cause, affecting 10–20% of all people with RP and the majority of those with X-linked RP. RPGR is located in photoreceptor connecting cilia. It interacts with a wide variety of ciliary proteins, but its exact function is unknown. Recently, there have been important advances both in our understanding of RPGR function and towards the development of a therapy. This review summarises the existing literature on human RPGR function and dysfunction, and suggests that RPGR plays a role in the function of the ciliary gate, which controls access of both membrane and soluble proteins to the photoreceptor outer segment. We discuss key models used to investigate and treat RPGR disease and suggest that gene augmentation therapy offers a realistic therapeutic approach, although important questions still remain to be answered, while cell replacement therapy based on retinal progenitor cells represents a more distant prospect.

## Highlights

• We discuss the proposed function(s) of the RPGR protein in photoreceptor maintenance.
• We highlight the animal models used in the field to research Rpgr.
• We discuss recent advances in gene therapy as a possible treatment for RPGR mutations.

## 1 Introduction

Photoreceptor degeneration is the hallmark of retinitis pigmentosa (RP), an inherited retinal dystrophy affecting 1 in 3000 people that commonly causes severe visual loss and blindness in middle life (Bramall et al., 2010; Wright et al., 2010; Sahel et al., 2014). Mutations in over 50 genes are now known to cause RP, which can be inherited as an autosomal dominant, autosomal recessive, X-linked or mitochondrial trait (https://sph.uth.edu/retnet/). There are syndromal and non-syndromal forms of RP and digenic forms have also been described (Kajiwara et al., 1994). Mutations in the *RPGR* gene account for 70–90% of the X-linked form of RP (XLRP) and 10–20% of all RP. Mutations in the *RP2* gene account for most of the remaining ∼20% of XLRP. XLRP is associated with a severe phenotype with marked rod and later cone cell death, extinguished rod electroretinogram and visual loss, commonly starting within the first few decades of life. At present, there is no treatment for disease caused by *RPGR* mutations (“RPGR disease”). However, an improved understanding of the protein's function has emerged in parallel with the emergence of novel technologies to model or potentially treat RPGR disease. Recent gene augmentation therapy successes using animal models have brought renewed hope for those affected by this debilitating illness (Bainbridge et al., 2008; Beltran et al., 2012).

## 2 RPGR structure and function

**Fig. 1:**
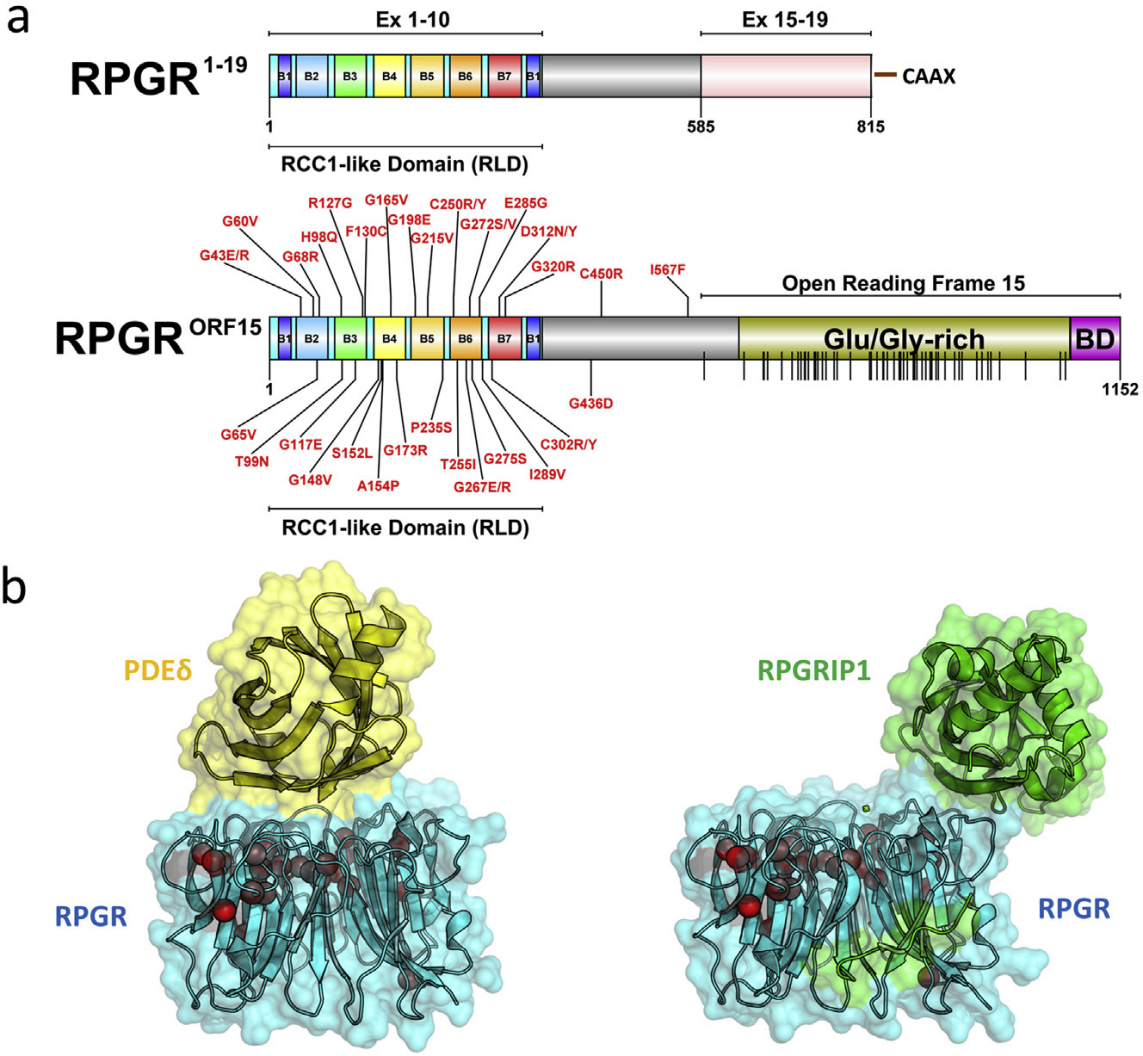
Major RPGR protein isoforms (constitutive RPGR^Ex1-19^ and RPGR^ORF15^) domain schematic. (a) Domain architecture schematics for both major isoforms are shown drawn to scale. The seven blades (B1 to B7) that form the beta-propeller RCC1-like domain (RLD) encoded within Exons 1–10 in both major isoforms are indicated. The RPGR^Ex1-19^ C-terminal isoprenylation site (CAAX) is shown. The location of the RPGR^ORF15^ Glutamate/Glycine-rich Domain and Basic Domain (BD) within the Open Reading Frame 15 are highlighted. All known disease-causing missense mutations (labelled), and a total of 52 known nonsense mutations specifically located within the Open Reading Frame 15 (vertical lines on domain schematic) are indicated. Mutation data was mapped from the Human Gene Mutation Database (Stenson et al., 2014) (accessed 27th May 2015). (b) The crystal structures of the RPGR RLD (blue) in complex with PDEδ (yellow) (Wätzlich et al., 2013) and RPGRIP1 (green) (Remans et al., 2014) are shown using PyMol (http://www.pymol.org) as surface representations with a transparency setting to highlight location of known missense mutations (red spheres, only alpha carbon atoms shown) on structure. PDEδ and RPGRIP1 interaction sites on the surface RPGR partially overlap (Remans et al., 2014).

The *RPGR* gene is located on the short arm of the X chromosome (Xp21.1) (Meindl et al., 1996; Vervoort et al., 2000) and expresses at least 10 alternative transcripts of which 5 are predicted to be protein coding (Kirschner et al., 1999; Roepman et al., 2000; Neidhardt et al., 2007; Schmid et al., 2010). Expression of the major splice variants (see below) is at least partly driven by a TATA-less proximal promoter (Shu et al., 2012), which fits with the widespread expression of *RPGR* in adult mammalian tissues. The promoter contains 4 transcriptional start sites which may influence expression in different tissues and within which the transcription factor SP1 was shown to activate *RPGR* transcription. The protein products of the two major human *RPGR* alternative transcripts have been extensively studied (Fig. 1a) (Meindl et al., 1996; Roepman et al., 1996; Vervoort et al., 2000; Mavlyutov et al., 2002; Hong et al., 2003; Patil et al., 2012a).

The first transcript to be identified was the ‘constitutive’ *RPGR*^Ex1-19^ isoform which is widely expressed in tissues (Meindl et al., 1996; Roepman et al., 1996; Vervoort et al., 2000) and is found within cells at the transition zone of primary and motile cilia (Hong et al., 2003; Iannaccone et al., 2003) or at centrosomes and their constituent centrioles in dividing cells. In the retina, the RPGR^Ex1-19^ isoform localises to the developing and mature photoreceptor connecting cilium (CC), connecting the inner and outer segments, but shows a slightly different developmental expression pattern and affinity for the axonemal (detergent insoluble) fraction compared with the other major RPGR isoform (RPGR^ORF15^), suggesting overlapping but also distinct functions (Wright et al., 2011). The RPGR^Ex1-19^ transcript encodes a predicted 90 kDa protein with 19 exons of the gene transcribed. Exons 1 to 10 of *RPGR*^Ex1-19^ encode an RCC1-like domain (Meindl et al., 1996; Wätzlich et al., 2013) and the C-terminus has an isoprenylation motif (CAAX), suggesting that this isoform is membrane bound, consistent with its reported attachment to endoplasmic reticulum membranes in addition to its presence at CC (Patil et al., 2012a). Yan et al. (1998) suggested a Golgi localisation for RPGR but this has not been confirmed (Patil et al., 2012a). No disease-causing mutations have been reported in exons 16 to 19. In contrast, all known mutations causing XLRP or related retinal dystrophies are found to affect the RPGR^ORF15^ isoform (Fig. 1a), which shows highest expression in the retina (Vervoort et al., 2000; Hong et al., 2003; Mavlyutov et al., 2002; Patil et al., 2012b; Iannaccone et al., 2003).

The human *RPGR*^ORF15^ transcript encodes a 1152 amino acid protein consisting of exons 1 to 14 of *RPGR*^Ex1-19^ followed by a unique C-terminal exon called ORF15, encoding 567 amino acids (Vervoort et al., 2000). Exon ORF15 is formed by exon 15 extending into intron 15 due to skipping of the splice donor site for exon 15 (Vervoort et al., 2000). The RPGR^ORF15^ isoform is predicted to be 127 kDa and exon ORF15 includes an acidic, repetitive, glutamic acid/glycine-rich domain and a basic C-terminal domain (Fig. 1a). The repetitive domain length is not under strict evolutionary constraint, varying considerably among species and across strains of mice and a partial truncation of murine ORF15 does not appear to alter its function (Vervoort et al., 2000; Hong et al., 2005). In contrast, the basic C-terminal domain is highly conserved among vertebrates (Shu et al., 2005). *RPGR*^*ORF15*^ is most strongly expressed in retina (Vervoort et al., 2000) where its protein product is localised to the photoreceptor CC (Hong et al., 2000, 2003; Mavlyutov et al., 2002). There is uncertainty as to whether or not RPGR^ORF15^ is present in the OS. Discrepancies between laboratories may be due to different antibodies, tissue processing procedures or species differences in OS structure (Mavlyutov et al., 2002; Shu et al., 2006). For example, it may depend on whether OS have multiple superficial incisures (as in humans and amphibia) or single deep incisures (as in rodent, bovine, canine OS) (Mavlyutov et al., 2002). However, while some antibodies clearly label human OS (Roepman et al., 2000; Iannaccone et al., 2003; Mavlyutov et al., 2002), this is not the case in rodents or pigs (Hong et al., 2003; Brunner et al., 2010; Wright et al., 2012) and the situation in bovine OS is unclear (Hong et al., 2003; Mavlyutov et al., 2002; Beltran et al., 2012). In dividing cells, RPGR^ORF15^ is present in centrosomes, while in non-dividing cells containing primary cilia it is found in the transition zone of the ciliary axoneme, the equivalent structure to the photoreceptor CC (Hong et al., 2003; Shu et al., 2005; Gakovic et al., 2011).

The exon ORF15 repetitive domain is a mutational hotspot for XLRP, accounting for two-thirds of all disease-causing mutations (Vervoort et al., 2000; Sharon et al., 2003) (Fig. 1a). Most ORF15 mutations are out-of-frame deletions of 1–5 bp that are predicted to produce truncated proteins with novel amino acid sequences and often a change from an acidic to a basic net charge (Wright and Shu, 2007). The basic C-terminal domain is predicted to be truncated or lost with most ORF15 mutations, which, in general, are associated with slightly milder disease than with mutations occurring in the N-terminal RCC1-like domain (Sharon et al., 2003). Indeed, the closer that exon ORF15 mutations are to the 3′ end, the less severe the phenotype. Mutations within a C-terminal exon, such as ORF15, are not expected to result in nonsense-mediated decay of the transcript so the presence of truncated, potentially gain-of-function, mutant proteins could exacerbate the loss of function due to premature stop codons (Fahim et al., 2011; Hong et al., 2004). This possibility is supported by the more severe phenotype accompanying a naturally occurring ORF15 mutation in XLPRA2 dogs which leads to an abnormally charged and novel C-terminus compared with an immediate truncation with the milder XLPRA1 phenotype in progressive retinal atrophy (PRA) dogs (Zeiss et al., 1999; Zhang et al., 2002). However, it is also possible that the XLPRA1 mutation is hypomorphic and the XLPRA2 mutation is null (Beltran et al., 2014).

## 3 RPGR in development

Analyses of *RPGR* knockout (KO) mice showed that RPGR is not essential for mammalian photoreceptor development (Hong et al., 2000). However, *RPGR*^Ex1-19^ and *RPGR*^ORF15^ isoforms have distinct developmental expression profiles in the murine retina. RPGR^Ex1-19^ is expressed early in development, declining as photoreceptors mature and *RPGR*^ORF15^ expression increases (Wright et al., 2011), suggesting a specific function, although there may be redundant mechanisms ensuring correct photoreceptor development. Zebrafish with an RPGR knockdown fail to develop OS and show systemic ciliary abnormalities, supporting the view that RPGR is required for normal retinal development in lower vertebrates (Shu et al., 2010; Ghosh et al., 2010). Further, the OS of XLPRA2 dogs are misaligned and fragmented prior to maturation (Beltran et al., 2006), although this might be secondary to the degeneration process. The role of RPGR in human retinal development is therefore unclear, but both vision and retinal structure appear to develop normally in patients with *RPGR* mutations. In contrast to its non-essential role during retinal development, RPGR has an essential function in the maintenance of mature photoreceptors.

## 4 RPGR interactions

**Fig. 2:**
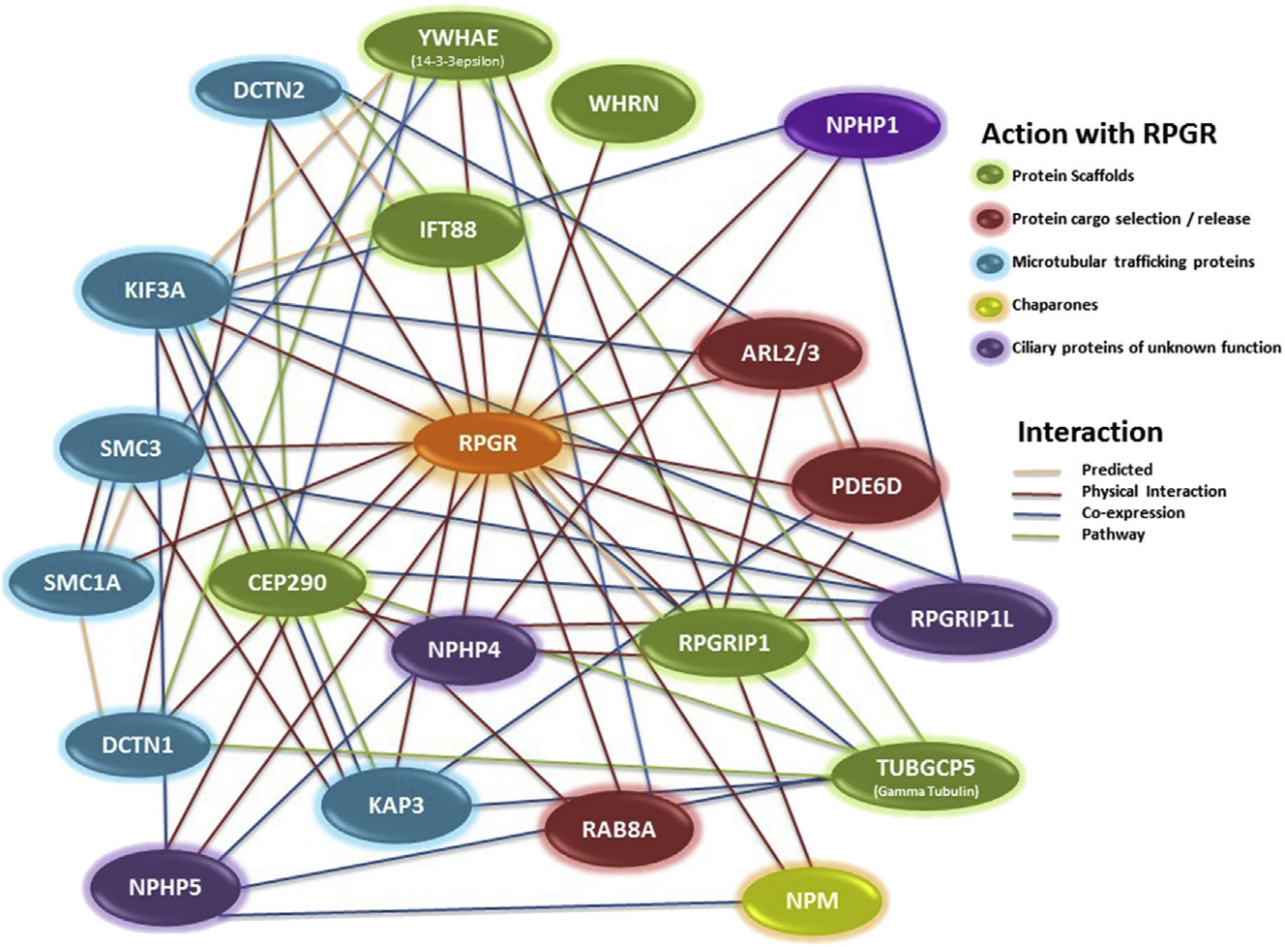
The RPGR Interactome. This complex and poorly understood network of cilia proteins has been classified on the basis of the existing literature, as discussed in this review. Links between proteins highlight the evidence in the literature for each interaction. The schematic was constructed using the STRING database (http://string-db.org) (Szklarczyk et al., 2015) and GeneMANIA (http://www.genemania.org/) (Warde-Farley et al., 2010).

A common approach to understanding the function of a protein is to characterise its interactions. Several RPGR-containing protein interactions and complexes have been proposed (Fig. 2). The emerging picture suggests that following its synthesis in the IS, RPGR is retained at the CC by binding to the RPGR interacting protein 1 (RPGRIP1), which was identified by yeast two-hybrid screening (Boylan and Wright, 2000; Roepman et al., 2000; Hong et al., 2001). RPGRIP1 has a coiled coil domain and three C2-like motifs that are found in many transition zone or CC proteins, either targeting these proteins to cell membranes or facilitating their interactions (Remans et al., 2014). The RPGRIP1 C-terminus RPGR interaction domain forms both homodimers and elongated filaments via interactions involving its coiled-coil and C-terminal domains (Zhao et al., 2003). RPGRIP1 is most strongly expressed in the CC of photoreceptors (Mavlyutov et al., 2002; Zhao et al., 2003; Castagnet et al., 2003) but is also present at the centrioles and basal bodies/transition zone of cultured cells (Shu et al., 2005). RPGRIP1 is essential for the localisation of RPGR to the CC (Zhao et al., 2003; Patil et al., 2012a; Li, 2014) and has one major retina-specific isoform, RPGRIP1α_1,_ which has been proposed to have a scaffolding function associated with a proposed “ciliary gate” and entry to the transition zone and fibres of primary cilia or photoreceptor CC (Remans et al., 2014). The transition zone contains Y-shaped fibres linking the axonemal microtubule doublets of the CC with the overlying plasma membrane, representing part of the proposed ciliary gate that restricts protein entry and exit to the OS (Reiter et al., 2012; Sung and Leroux, 2013; Rachel et al., 2012). The localisation of RPGRIP1 to the CC is in turn dependent on another ciliary protein, SPATA7, in which mutations result in rhodopsin mislocalisation to the plasma membrane (8-fold increase), IS (5-fold increase) and outer nuclear layer. *SPATA7* mutations cause the severe early-onset retinopathy Leber congenital amaurosis (LCA, type 3) and juvenile RP (Eblimit et al., 2015). Mutations in *RPGRIP1* also cause LCA (type 6) (Dryja et al., 2001; Gerber et al., 2001) as well as cone-rod dystrophy (CORD13) (Hameed et al., 2003). A recently generated complete *RPGRIP1* KO mouse produces ‘naked cilia’ which fail to form OS and shows mislocalisation of rod and cone opsins (although contradicted by Patil et al., 2012b) as well as other OS proteins, indicating a role both in disc morphogenesis and OS formation (Won et al., 2009; Patil et al., 2012b). A partial *RPGRIP1* knockout mouse showed disorganised OS with elongated discs, partially mislocalised rod and cone opsins and normal CC, but a severe early-onset retinal degeneration also resembling LCA (Zhao et al., 2003).

**Fig. 3:**
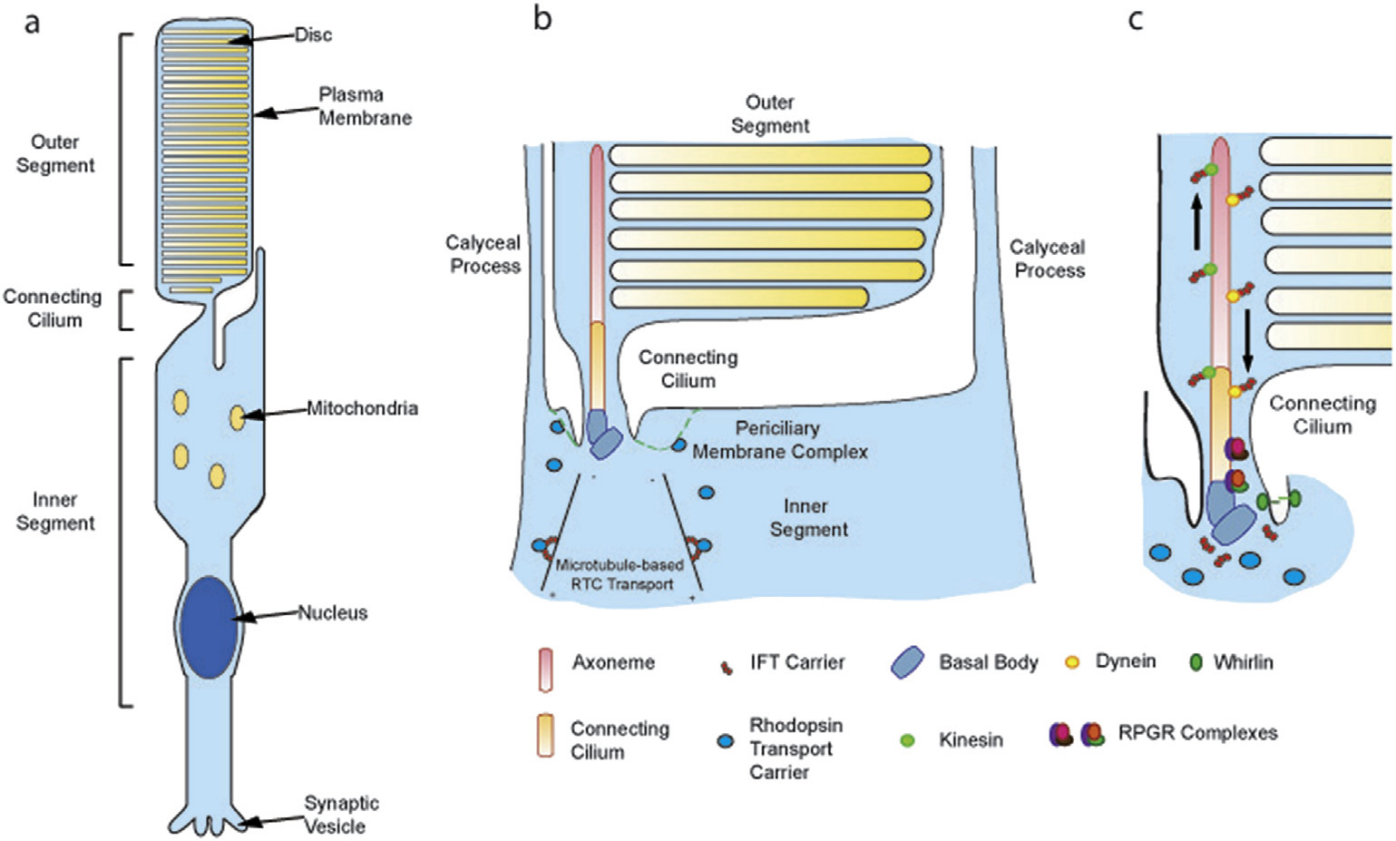
The Photoreceptor connecting cilium. A modified sensory cilium, comprising the axoneme, connecting cilium (CC) and basal bodies (BB), links the inner and outer segment in vertebrate photoreceptors. (a) Microtubule transport facilitates Rhodopsin Transport Carrier (RTC) delivery to the pericilliary membrane complex (green dotted line in (b), at the base of the BBs, prior to movement into the CC. The BBs co-ordinate microtubule assembly whilst the non-motile axoneme (comprising nine microtubule doublets) acts as a backbone and facilitates intra-flagellar transport (IFT) up and down the CC. (c) The CC serves to compartmentalise the photosensitive pigment rhodopsin into stacks of discs that fill the outer segment. The CC is therefore crucial for maintenance and survival of the photoreceptor. The diagram was modified from Maerker et al. (2008).

RPGR has also been implicated in the trafficking or quality control of membrane proteins moving to/from the OS, since rod and cone opsins are mislocalised to the IS or plasma membrane in a variety of CC transport mutants (e.g. kinesin-2, intraflagellar transport or IFT proteins) and RP/LCA mouse models (*Bbs2*, *Ahi1*, *Rp1*, *Rpgrip1*, *Cep290* mice), including several RPGR disease models. The latter include a naturally occurring *Rpgr* mutant mouse (*rd9*; Thompson et al., 2012), two gene targeted mouse models, namely *Rpgr* KO mice (Hong et al., 2000) and Rpgr^ΔEx4^ mice (Brunner et al., 2010), XLPRA1 mutant dogs (Zhang et al., 2002) and two human XLRP carriers with *RPGR* mutations (Adamian et al., 2006; Aguirre et al., 2002). Transport of opsin-containing vesicles from Golgi to the OS minimally requires a rhodopsin C-terminal targeting motif (VxPx), binding to a dynein motor protein subunit (Tctex-1), vesicle docking at the base of the CC, and (by analogy with protist cilia) loading onto IFT complexes (e.g. complex B subunit Ift20) (Keady et al., 2011). Docking of rhodopsin carrier vesicles probably occurs at the periciliary membrane complex, a specialised apical membrane microdomain directly facing the CC (Fig. 3). Further transport to the CC and nascent discs requires (again by analogy with protists) another IFT complex (e.g. complex A subunit Ift40) and the kinesin-2 motor (Crouse et al., 2014; Keady et al., 2011). Defects in transport between the membrane docking and CC delivery steps should result in rhodopsin accumulation in the OS plasma membrane, as seen in several models (e.g. Spata7^−/−^ mutants). In contrast, mutants that are defective in vesicular transport of opsins (e.g. BBS proteins, TULP1) show accumulation of vesicles near the base of the IS while mutants with absent OS (e.g. Rho^−/−^ mice) show vesicle accumulation at the distal tip of the CC, neither of which were found in Rpgrip1, Spata7 or Rpgr KO mice (Hong et al., 2000; Won et al., 2009). In addition, while opsins were mislocalised prior to the onset of apoptosis, OS disk or shuttling proteins PRPH2, ROM-1 and transducin were all correctly localised in the Spata7 KO mice, arguing for a specific opsin transport defect in these mice with presumed abrogation of the Spata7-RPGRIP1-RPGR protein complex (Eblimit et al., 2015). It has been argued that discrepancies in observing rhodopsin mislocalization in some animal models of inherited retinal degeneration may be attributed to variability in the stages of photoreceptor degeneration at the time of analysis. Indeed, due to the abundance of rhodopsin, its mislocalisation to the inner segment will inevitably occur once outer segment degeneration begins, in which case it would be a secondary consequence rather than a primary cause of disease. However, several *RPGR* disease models demonstrate opsin mislocalisation prior to any discernible photoreceptor degeneration (Hong et al., 2000; Thompson et al., 2012).

There is indirect molecular evidence linking RPGR function with vesicle trafficking, for example RPGR interactions with RAB8, whirlin and the cytoskeleton (see below), but since proposed ciliary gate proteins such as RPGR, RPGRIP1 and CEP290 are all required for opsin localisation to the OS it is currently more plausible that this defect is secondary to defective ciliary gate functions.

Recent work has gone some way towards elucidating the RPGRIP1 interaction with RPGR by showing that the RPGRIP1 interaction domain of RPGR partially overlaps with the domain interacting with PDEδ (PDE6D) (Wätzlich et al., 2013, Remans et al., 2014) (Fig. 1b), a highly evolutionarily conserved prenyl binding protein that also binds RPGR (Linari et al., 1999). PDEδ interacts with a variety of prenylated G proteins and phototransduction proteins (Baehr, 2014). RPGRIP1 may compete with and weaken PDEδ binding to its cargo, following RPGR-mediated PDEδ delivery to the CC, perhaps indicating a cargo delivery or sorting role (Remans et al., 2014). The 3-dimensional (3-D) structure of the RPGR RCC1-like domain indicates that it binds PDEδ at a highly sequence-conserved surface patch (Fig. 1b). Binding of PDEδ to cargo proteins is in turn regulated by two unprenylated G proteins, ARL2 and ARL3, which are involved in cargo release (Wätzlich et al., 2013). The *RP2* gene product greatly accelerates the hydrolysis of GTP-bound ARL3. *RP2* null mutations are therefore thought to impair the trafficking of prenylated proteins to the photoreceptor OS (Baehr, 2014). A rare null mutation in the *PDE6D* gene causes the ciliopathy Joubert syndrome, associated with retinal dysplasia and microphthalmia (Thomas et al., 2014). A PDEδ knockout mouse develops a more subtle phenotype with a slowly progressive rod-cone dystrophy together with mislocalisation of prenylated phototransduction proteins such as rhodopsin kinase (GRK1) and the catalytic subunits of rod and cone cyclic GMP phosphodiesterase (PDE6) (Zhang et al., 2007). Non-prenylated proteins such as rhodopsin were correctly localised to the OS, in contrast to the situation with *RPGR* mutations (Zhang et al., 2007). Despite the apparent phenotypic differences between mouse and human (which may reflect mutational differences), the results overall seem to indicate the involvement of RPGR in multiple trafficking, gatekeeping and/or cargo delivery steps required for outer segment function and maintenance.

Another RPGR-interacting protein is CEP290 (NPHP6), which localises to centrosomes throughout the cell cycle and to the photoreceptor CC in mouse photoreceptors (Chang et al., 2006). It was proposed that CEP290 has a role in microtubule nucleation, centrosome and cilia formation. CEP290 interacts directly with RPGR^ORF15^ and both proteins form part of a microtubule-associated protein complex at the centrosome (Chang et al., 2006). Experiments conducted in the model organism *Chlamydomonas reinhardtii* provide compelling evidence that CEP290 is a dynamic component of the transition fibres and Y-linkers that are thought to perform a ciliary gate function, since *CEP290* mutants alter the composition of several mainly soluble flagellar proteins (Craige et al., 2010). RPGR is also mislocalised from the CC to the IS in a naturally occurring *Cep290* mouse mutant (*rd16*), suggesting that RPGR is also involved in sorting/loading of specific IFT and other protein cargoes at this site (Chang et al., 2006; Craige et al., 2010).

CEP290 has numerous interacting partners (Fig. 2) and *CEP290* mutations are associated with retinal degeneration in six partially overlapping ciliopathy syndromes, consistent with a central role in ciliary and/or outer segment maintenance (Joubert Syndrome (JBS), nephronophthisis (NPHP), Leber congenital amaurosis (LCA), Senior-Loken syndrome (SLS), Meckel-Gruber syndrome (MKS) and Bardet-Biedl syndrome (BBS) (Sayer et al., 2006)). In summary, CEP290, RPGR and RPGRIP1 are each associated with ciliary gate functions that regulate protein trafficking to/from the OS.

RPGR also interacts with Structural Maintenance of Chromosome (SMC) proteins SMC1 and SMC3 (Khanna et al., 2005), whose functions are thought to include assembly of microtubular spindle poles during mitosis. The same study also showed that RPGR^ORF15^ co-immunoprecipitates with a variety of basal body (14-3-3ε, γ-tubulin, IFT88) and both anterograde and retrograde microtubular transport proteins (kinesin II subunits KIF3A, KAP3 and dynein intermediate and heavy chains, dynactin subunits DCTN1 and DCTN2), suggesting a role in CC trafficking although whether as a cargo or a regulator is unclear.

Several members of the nephrocystin family of proteins interact with RPGR. The nephrocystins are a group of proteins that localise to primary cilia in kidney and in many cases to photoreceptor CC (Rachel et al., 2012). They are associated with the renal disease nephronophthisis (NPHP), a medullary cystic disease. In 10% of cases, nephrocystins also cause syndromal forms of NPHP with retinal degeneration (e.g. SLS and JBS). RPGR interacts with the CC proteins NPHP1 and NPHP4 (Murga-Zamalloa et al., 2010a) in distinct complexes, as well as with NPHP6 (CEP290) discussed above. RPGRIP1α is required for the ciliary localisation of NPHP4, RPGR and another interacting protein, SDCCAG8 (Patil et al., 2012b). Proteomic analyses suggest the existence of two major complexes that potentially include RPGR, NPHP1-2-4-8/RPGRIP1L1, and NPHP2-5-6/CEP290 (Sang et al., 2011). The RPGR-NPHP1/4 binding sites overlap, so a ‘hand over’ mechanism was proposed, perhaps facilitating efficient cargo delivery to the CC. Mutations in *NPHP5*/*IQCB1*, encoding an IQ-domain protein which localises to the photoreceptor CC, cause the ciliopathy SLS. An interaction between NPHP5 and RPGR^ORF15^ has been demonstrated (Otto et al., 2005) and the NPHP5 interaction with CEP290 appears crucial for ciliogenesis (Barbelanne et al., 2013). NPHP5 regulates the multi-subunit BBSome complex, located at the basal bodies and centriolar satellites, which is thought to be concerned with trafficking of membrane cargoes to the CC (Barbelanne et al., 2015). In short, NPHP1, 4, 5, 6 (CEP290) and 8 (RPGRIP1L1) all interact with RPGR, and while their precise functions are unclear, they appear to influence the assortment and trafficking of cargoes through the CC to the OS, as well as through the ciliary transition zone in renal medullary cells.

The RCC1-like domain of RPGR was initially predicted to act as a guanine nucleotide exchange factor (GEF) for a Ran-like GTPase (Meindl et al., 1996). RAB8 is a GTPase that shuttles rhodopsin transport carriers to the CC base so that its inhibition leads to mislocalisation of rhodopsin (Moritz et al., 2001). Rabin8 is its primary GEF (Hattula et al., 2002) but RPGR has also been reported to activate RAB8 (Murga-Zamalloa et al., 2010b). Human *RPGR* mutations perturb this and *RPGR* knock-down in cells mislocalises RAB8 away from primary cilia, suggesting that it facilitates RAB8-led rhodopsin trafficking. However, only one residue required for RCC1 GEF function is conserved in RPGR (Renault et al., 1998) and the β-hairpin extension required for GEF activity is not found in RPGR (Wätzlich et al., 2013), raising questions as to its role in activating RAB8, so this finding needs further corroboration.

The *RPGR* exon ORF15 repeat domain is a hotspot for disease but its function is unclear. It is predicted to be unstructured and, so far, interacting partners have not been identified. In contrast, the basic C-terminal domain (Fig. 1a) is highly conserved across vertebrates and binds nucleophosmin (NPM), a protein chaperone (Shu et al., 2005). The role of this interaction is unknown since, although it partially co-localises with RPGR at centrosomes during metaphase, in photoreceptors it localises to the nucleoli. RPGR has been seen in the nuclei in some cell types but not in photoreceptors (Lu et al., 2005). Several other ciliary proteins, including CEP290, can also be found in nuclei, which is perhaps relevant to recent findings linking both renal and retinal ciliopathies with DNA damage response proteins, such as NEK8, ATR, ZNF423 and CEP164, which are often located at centrosomes, primary cilia or nuclear DNA damage foci (Chaki et al., 2012; Choi et al., 2013; Jackson, 2013; Valdés-Sánchez et al., 2013).

Interestingly, the ORF15 basic domain also interacts with Whirlin (WHRN), a scaffold protein expressed in cochlear hair cells and photoreceptors (Wright et al., 2012). WHRN has a role in cytoskeletal assembly both in inner ear stereocilia (Mburu et al., 2006; van Wijk et al., 2006) and in photoreceptors, where its interaction with the actin cross-linking protein espin is important in regulating the actin filament network in the periciliary membrane complex, defined by the presence of the proteins usherin, whirlin or VLGR1 (Peters et al., 1983; Yang et al., 2010) (Fig. 3). An actin bundle appears to connect the periciliary membrane complex with the basal body, along which the actin-based motor protein myosin VIIA appears to travel (Williams et al., 1988; Wang et al., 2012). N-terminal mutations in WHRN cause Usher syndrome (type 2D), a syndromic form of RP associated with non-congenital sensorineural deafness. This links RPGR^ORF15^ to the Usher protein network, which is in turn thought to directly link the CC with the periciliary membrane complex, where post-Golgi vesicles are proposed to dock and sort their cargoes (Kremer et al., 2006; Maerker et al., 2008) (Fig. 3).

The Usher protein network shows clear links to the actin cytoskeleton (Kremer et al., 2006) and RPGR knockdown leads to stronger expression of actin stress fibres (Gakovic et al., 2011). Actin regulates vesicle trafficking and its polymerisation inhibits ciliogenesis, whilst depolymerisation doubles cilia length (Kim et al., 2010) and induces elongated, nascent discs in photoreceptors (Vaughan and Fisher, 1989) reminiscent of the *Rpgrip1* KO mouse reported by Zhao et al., 2003. Actin is also localised to the distal portion of the CC where discs form (Chaitin and Burnside, 1989) and provides the constricting forces required to facilitate membrane scission (Knödler et al., 2010). RAB8-driven vesicle trafficking occurs along microtubules but also appears to be actin-dependent (Hattula et al., 2006; Deretic et al., 1995). Increased actin branching inhibits rhodopsin transport by inhibiting RAB8 activation and localisation to the OS (Deretic et al., 1995; Moritz et al., 2001). RPGR may therefore influence actin regulation of rhodopsin transport carrier sorting to the CC, disc budding and/or completion of disc formation.

In summary, RPGR is localized to the photoreceptor CC and to the corresponding structures (transition zone) in primary cilia, as a result of its interaction with one or more RPGRIP1 isoforms. RPGR^ORF15^ functions are likely to be involved in some aspect(s) of the ciliary gate, and trafficking or sorting of cargoes, some of which originate from the periciliary membrane complex. Whether this function is mediated by RPGRIP1, PDEδ, nephrocystins, RAB8, WHRN, the actin or microtubule based cytoskeleton, or a combination of these, remains to be resolved.

## 5 Human RPGR disease

Human RPGR disease is a severe form of retinal degeneration, leaving patients visually impaired at a relatively young age. There is significant phenotypic variability between XLRP patients with RPGR disease. RCC1-like domain (RLD) mutations tend to cause more severe disease than ORF15 mutations (Sharon et al., 2003) and some are also implicated in systemic ciliary disease. RLD mutations have been subdivided on the basis of their location in the protein and effects on function into six classes (Patil et al., 2012a). The classes considered RPGR protein folding, stability and interactions with RPGRIP1α_1_ or PDEδ. More recently, high-resolution 3-D structural data for the RPGR RCC1-like domain in complex with PDEδ (Wätzlich et al., 2013) and RPGRIP1 (Remans et al., 2014) was determined by crystallography. These structures revealed that previously described missense mutations were not located at the binding site in the case of RPGR-PDEδ, and likewise those mutations located in the vicinity of the RPGR-RPGRIP1 binding site did not perturb this interaction when tested biochemically. Several known patient missense mutations were however likely to impact on the structural integrity of the beta-propeller fold (Wätzlich et al., 2013).

Clinical diagnoses in RPGR disease vary between classical XLRP (95%), cone dystrophy, cone-rod dystrophy or atrophic macular degeneration (3%) and ciliopathy (2%) (Dry et al., 1999; Zito et al., 2003; Iannaccone et al., 2003; Shu et al., 2007). Further, dizygotic twins with a single nucleotide deletion in ORF15 (1339delA) were shown to be discordant for disease severity (Walia et al., 2008) and large families can display marked phenotypic variability (Fahim et al., 2011; Huang et al., 2012). In addition to varying degrees of residual activity, clinical variability may be due to environmental influences, stochastic developmental effects and genetic background (epistasis), where *RPGR* mutations are affected by 'modifier genes.' Recently a SNP screen of *RPGR* patients displaying varying disease severity showed that SNPs in *IQCB1* and *RPGRIP1L* were associated with disease severity (Fahim et al., 2011). Until XLRP patients have their whole genome routinely sequenced it will be hard to evaluate whether such disease modifiers are present, which could influence prognosis or patient selection for trials of emerging therapies (see below).

Several studies have examined RPGR disease progression. Genotype-phenotype concordance has been demonstrated regarding electrophysiology (rod-cone versus cone-rod dystrophy) and field loss patterns (Zahid et al., 2013). Interestingly, 3′-end ORF15 mutations cause cone-rod disease on ERG analysis, indicating relative sparing of rods (Sharon et al., 2003; Zahid et al., 2013). Analysis of disease progression over time showed a steady deterioration in visual acuity and fields (Huang et al., 2012) with two main field loss patterns; most commonly, a mid-peripheral scotoma separating a preserved central cone island from a region of preserved peripheral rod function and, less commonly, a paracentral loss leaving small central islands of cone function which could be maintained until late in disease. In teenage patients, rod function varied from normal to profound loss but deteriorated steadily thereafter. Cone dysfunction was milder and showed less variability. Female *RPGR* carriers are generally asymptomatic but can display clinical abnormalities with tapetal reflexes, peripheral retinal thinning and severe rod and cone dysfunction (Bird, 1975; Acton et al., 2013). This highlights the importance of examining female relatives, particularly regarding genetic counselling.

## 6 Models of RPGR disease

### 6.1 Animal models

Various *RPGR* disease models have been studied. RPGR knockdown in ciliated cell lines is one approach that has helped to unravel disease mechanisms (e.g. Gakovic et al., 2011). Animal models have also been used. In the original *Rpgr* KO mouse, the retina developed normally but showed degenerative changes by 2 months (Hong et al., 2000). Cone opsins (but not rhodopsin) were mislocalised at an early stage. By 6.5 months, photoreceptor function was compromised and discs appeared disorganised despite a normal CC, suggesting trafficking and/or disc membrane formation abnormalities. This relatively mild phenotype resembles late-onset cone-rod degeneration and subsequent analysis reported residual RPGR^ORF15^ expression (Khanna et al., 2005). A recent *Rpgr* exon1 conditional knockout mouse on a different (BALB/c) genetic background showed a faster rate of retinal degeneration and visual loss than the *Rpgr* KO mouse (Huang et al., 2012). A naturally occurring *rd9* mouse was found to have a 32-bp duplication in ORF15, producing a much slower degeneration (Thompson et al., 2012). Different strains of mice sharing the same *RPGR* mutation can express a different phenotype, highlighting the role of genetic background effects (Brunner et al., 2010). *Rpgr*^ORF15^ overexpression partially rescues the *Rpgr* KO mouse, suggesting a loss-of-function effect of the protein (Hong et al., 2005). However, overexpression of a truncated murine-specific ORF15 variant led to more rapid degeneration compared to the KO alone, on both wild-type and *Rpgr*-null backgrounds (Hong et al., 2004), suggesting a gain-of-function (GOF) role for this particular variant. A GOF phenotype is difficult to reconcile with clinical disease, since most female carriers remain asymptomatic. Carrier females are usually protected by X chromosome inactivation and/or cell autonomous mutational effects but the substantial rescue of the *Rpgr* KO mouse and XLPRA dogs (see below) by gene augmentation therapy also argues against significant GOF mutations in XLRP patients.

Two naturally occurring *RPGR* disease models exist in dogs. Canine X-linked progressive retinal atrophy (XLPRA) occurs in the Siberian Samoyed husky (XLPRA1; 5-bp deletion in exon ORF15) and in a mixed breed dog (XLPRA2; 2-bp deletion in exon ORF15). The XLPRA1 mutation allows normal photoreceptor development and function until 6 months of age followed by a slow degeneration of rods, which die by apoptosis. The XLPRA2 phenotype is severe, with abnormal retinal development leading to disorganised OS and rapid degeneration (Zeiss et al., 1999; Zhang et al., 2002; Beltran et al., 2006). These dogs are excellent large animal models and provide a stepping-stone towards clinical trials for novel therapies (see below). Finally, a zebrafish knockdown model of RPGR disease has been reported to show ciliary abnormalities (Shu et al., 2010). Animal models have drawbacks, not the least of which is their cost. Alternative technologies to supplement these models may therefore be useful.

### 6.2 iPSC technology and three-dimensional retinal culture

The prospect of reprogramming terminally-differentiated somatic cells from adult tissue into pluripotent cells was demonstrated in principle by the cloning of tadpoles and sheep (Gurdon, 1962; Wilmut et al., 1997) and has been realised in humans (Takahashi et al., 2007). These induced pluripotent stem cells (iPSCs) can be derived from any genetic background, including patients with *RPGR* disease, so ‘disease-in-a-dish’ modelling is possible if mature photoreceptors can be derived from iPSCs.

Major progress has been made in pattering stem cells to produce post-mitotic photoreceptors. Exogenous molecules promote such conversion by means of many published protocols (Lamba et al., 2006; Osakada et al., 2008; Mellough et al., 2012). Initially these protocols encouraged two-dimensional culture, but recent understanding of the importance of the extracellular matrix in recapitulating endogenous signalling required for human retinal development (Nakano et al., 2012) has led biologists to replace 2D modelling with 3D protocols, with improved results. Floating aggregate cultures facilitate organised, stratified neuroretina production with light-sensitive photoreceptors being generated (Zhong et al., 2014). These cultures provide an excellent model of *RPGR* disease.

## 7 Future treatment options

### 7.1 Gene therapy

Gene augmentation therapy appears a feasible, safe treatment strategy for at least some inherited retinal dystrophies (Bainbridge et al., 2008; Maguire et al., 2008; Cideciyan, 2010; Jacobson et al., 2012). Recently, there was progress towards *RPGR* gene augmentation therapy when an adeno-associated virus 2/5 vector (packaging restriction < 4.7 kb) mediated the transfer of full-length human *RPGR*^ORF15^ (driven by the human IRBP promoter) into photoreceptors, preventing degeneration in both canine *RPGR* disease models and resulting in increased numbers of photoreceptors, preserved structure and absence of rhodopsin and L/M cone opsin mislocalization (Beltran et al., 2012). Reduced Muller cell reactivity in treated eyes also indicated that the harmful retinal remodelling found in this disease was also reduced. However, with RPE65-LCA patients, gene therapy could not prevent retinal degeneration progressing over a three year period despite substantial visual improvement at first (Cideciyan et al., 2013). Similarly with the canine model of *RPGR* disease, degeneration continued unless treatment was initiated prior to photoreceptor loss (Cideciyan et al., 2013). Improvement in visual function therefore cannot be assumed to imply protection from degeneration, suggesting the need for a combinatorial approach in treating retinal dystrophies. Further caution comes from the finding that overexpression of *Rpgr*^Ex1-19^ (but not *Rpgr*^ORF15^) on an *Rpgr* null background led to a more severe phenotype than in the *Rpgr* null mouse (Wright et al., 2011). While over-expressing wildtype *Rpgr*^ORF15^ is better tolerated, this may still cause problems by altering Rpgr isoform ratios. Overexpression of a genomic fragment containing the entire mouse *Rpgr* gene resulted in flagellar defects and male infertility, with a severity correlating with *Rpgr* copy number (Brunner et al., 2008). Rpgr co-localised with acetylated α-tubulin in mouse sperm flagella (Khanna et al., 2005). These results suggest the need for careful control of *RPGR* expression levels.

### 7.2 Cell replacement therapy

Advances in stem cell-derived retinal differentiation has led to the possibility of photoreceptor precursor transplant into diseased eyes. Embryonic stem cell (ESC)-derived retinal pigment epithelium (RPE) for cell replacement in RPE dystrophies is already in clinical trials and appears to be safe (Schwartz et al., 2015). The optimum developmental stage of photoreceptor progenitor cells for transplantation has been established (MacLaren et al., 2006) and the procedure has led to improvement of vision in blind mice (Pearson et al., 2012; Barber et al., 2013; Singh et al., 2013). Photoreceptor progenitor cells derived from three-dimensional ESC cultures can also integrate into rodent retina (Gonzalez-Cordero et al., 2013). However, whilst cell replacement may be a viable treatment option for retinal dystrophies in the future, the extent of rod loss experienced in *RPGR* disease raises questions as to whether sufficient numbers of rod progenitors can integrate into the retina to make an impact on visual loss.

## 8 Conclusion

*RPGR* mutations are responsible for 10–20% of all RP patients and cause severe disease for which there is no treatment. This review has sought to summarise current understanding of RPGR biology, including proposed roles in the ciliary gate that regulates protein trafficking to and from the photoreceptor OS. The results of treating animal models of *RPGR* disease show considerable promise and suggest that gene replacement therapy and, in the future, cell replacement therapy, could lead to improved visual function in this disorder.

## References

[bib1] Acton J.H., Greenberg J.P., Greenstein V.C., Marsiglia M., Tabacaru M., Theodore Smith R., Tsang S.H. (2013). Evaluation of multimodal imaging in carriers of X-linked retinitis pigmentosa. Exp. Eye Res..

[bib2] Adamian M., Pawlyk B.S., Hong D.H., Berson E.L. (2006). Rod and cone opsin mislocalization in an autopsy eye from a carrier of X-linked retinitis pigmentosa with a Gly436Asp mutation in the RPGR gene. Am. J. Ophthalmol..

[bib3] Aguirre G.D., Yashar B.M., John S.K., Smith J.E., Breuer D.K., Hiriyanna S., Swaroop A., Milam A.H. (2002). Retinal histopathology of an XLRP carrier with a mutation in the RPGR exon ORF15. Exp. Eye Res..

[bib4] Baehr W. (2014). Membrane protein transport in photoreceptors: the function of PDEδ: the Proctor lecture. Invest Ophthalmol. Vis. Sci..

[bib5] Bainbridge J.W., Smith A.J., Barker S.S., Robbie S., Henderson R., Balaggan K., Viswanathan A., Holder G.E., Stockman A., Tyler N., Petersen-Jones S., Bhattacharya S.S., Thrasher A.J., Fitzke F.W., Carter B.J., Rubin G.S., Moore A.T., Ali R.R. (2008). Effect of gene therapy on visual function in Leber's congenital amaurosis. New. Engl. J. Med..

[bib6] Barbelanne M., Song J., Ahmadzai M., Tsang W.Y. (2013). Pathogenic NPHP5 mutations impair protein interaction with Cep290, a prerequisite for ciliogenesis. Hum. Mol. Genet..

[bib7] Barbelanne M., Hossain D., Chan D.P., Peränen J., Tsang W.Y. (2015). Nephrocystin proteins NPHP5 and Cep290 regulate BBSome integrity, ciliary trafficking and cargo delivery. Hum. Mol. Genet..

[bib8] Barber A.C., Hippert C., Duran Y., West E.L., Bainbridge J.W., Warre-Cornish K., Luhmann U.F., Lakowski J., Sowden J.C., Ali R.R., Pearson R.A. (2013). Repair of the degenerate retina by photoreceptor transplantation. Proc. Natl. Acad. Sci..

[bib9] Beltran W.A., Hammond P., Acland G.M., Aguirre G.D. (2006). A frameshift mutation in RPGR exon ORF15 causes photoreceptor degeneration and inner retina remodeling in a model of X-linked retinitis pigmentosa. Invest Ophthalmol. Vis. Sci..

[bib10] Beltran W.A., Cideciyan A.V., Lewin A.S., Iwabe S., Khanna H., Sumaroka A., Chiodo V.A., Fajardo D.S., Román A.J., Deng W.T., Swider M., Alemán T.S., Boye S.L., Genini S., Swaroop A., Hauswirth W.W., Jacobson S.G., Aguirre G.D. (2012). Gene therapy rescues photoreceptor blindness in dogs and paves the way for treating human X-linked retinitis pigmentosa. Proc. Natl. Acad. Sci. U. S. A..

[bib11] Beltran W.A., Cideciyan A.V., Lewin A.S., Hauswirth W.W., Jacobson S.G., Aguirre G.D. (2014). Gene augmentation for X-linked retinitis pigmentosa caused by mutations in RPGR. Cold Spring Harb. Perspect. Med..

[bib12] Bird A.C. (1975). X-linked retinitis pigmentosa. Br. J. Ophthalmol..

[bib13] Boylan J., Wright A.F. (2000). Identification of a novel protein interacting with RPGR. Hum. Mol. Genet..

[bib14] Bramall A.N., Wright A.F., Jacobson S.G., McInnes R.R. (2010). The genomic, biochemical, and cellular responses of the retina in inherited photoreceptor degenerations and prospects for the treatment of these disorders. Annu. Rev. Neurosci..

[bib15] Brunner S., Colman D., Travis A.J., Luhmann U.F., Shi W., Feil S., Imsand C., Nelson J., Grimm C., Rülicke T., Fundele R., Neidhardt J., Berger W. (2008). Overexpression of RPGR leads to male infertility in mice due to defects in flagellar assembly. Biol. Reprod..

[bib16] Brunner S., Skosyrski S., Kirschner-Schwabe R., Knobeloch K.P., Neidhardt J., Feil S., Glaus E., Luhmann U.F., Rüther K., Berger W. (2010). Cone versus rod disease in a mutant Rpgr mouse caused by different genetic backgrounds. Invest Ophthalmol. Vis. Sci..

[bib17] Castagnet P., Mavlyutov T., Cai Y., Zhong F., Ferreira P. (2003). RPGRIP1s with distinct neuronal localization and biochemical properties associate selectively with RanBP2 in amacrine neurons. Hum. Mol. Genet..

[bib18] Chaitin M., Burnside B. (1989). Actin filament polarity at the site of rod outer segment disk morphogenesis. Invest Ophthalmol. Vis. Sci..

[bib19] Chaki M., Airik R., Ghosh A.K., Giles R.H., Chen R., Slaats G.G., Wang H., Hurd T.W., Zhou W., Cluckey A., Gee H.Y., Ramaswami G., Hong C.J., Hamilton B.A., Cervenka I., Ganji R.S., Bryja V., Arts H.H., van Reeuwijk J., Oud M.M., Letteboer S.J., Roepman R., Husson H., Ibraghimov-Beskrovnaya O., Yasunaga T., Walz G., Eley L., Sayer J.A., Schermer B., Liebau M.C., Benzing T., Le Corre S., Drummond I., Janssen S., Allen S.J., Natarajan S., O'Toole J.F., Attanasio M., Saunier S., Antignac C., Koenekoop R.K., Ren H., Lopez I., Nayir A., Stoetzel C., Dollfus H., Massoudi R., Gleeson J.G., Andreoli S.P., Doherty D.G., Lindstrad A., Golzio C., Katsanis N., Pape L., Abboud EB,Al-Rajhi A.A., Lewis R.A., Omran H., Lee E.Y., Wang S., Sekiguchi J.M., Saunders R., Johnson C.A., Garner E., Vanselow K., Andersen J.S., Shlomai J., Nurnberg G,Nurnberg P., Levy S., Smogorzewska A., Otto E.A., Hildebrandt F. (2012). Exome capture reveals ZNF423 and CEP164 mutations, linking renal ciliopathies to DNA damage response signaling. Cell.

[bib20] Chang B., Khanna H., Hawes N., Jimeno D., Lillo C., Parapuram S.K., Cheng H., Scott A., Hurd R.E., Sayer J.A., Otto E.A., Attanasio M., O'Toole J.F., Jin G., Shou C., Hildebrandt F., Williams D.S., Heckenlively J.R., Swaroop A. (2006). In-frame deletion in a novel centrosomal/ciliary protein CEP290/NPHP6 perturbs its interaction with RPGR and results in early-onset retinal degeneration in the rd16 mouse. Hum. Mol. Genet..

[bib21] Choi H.J., Lin J.R., Vannier J.B., Slaats G.G., Kile A.C., Paulsen R.D., Manning D.K., Beier D.R., Giles R.H., Boulton S.J., Cimprich K.A. (2013). NEK8 links the ATR-regulated replication stress response and S phase CDK activity to renal ciliopathies. Mol. Cell..

[bib22] Cideciyan A.V. (2010). Leber congenital amaurosis due to RPE65 mutations and its treatment with gene therapy. Prog. Retin Eye Res..

[bib23] Cideciyan A.V., Jacobson S.G., Beltran W.A., Sumaroka A., Swider M., Iwabe S., Roman A.J., Olivares M.B., Schwartz S.B., Komáromy A.M., Hauswirth W.W., Aguirre G.D. (2013). Human retinal gene therapy for Leber congenital amaurosis shows advancing retinal degeneration despite enduring visual improvement. Proc. Natl. Acad. Sci. U. S. A..

[bib24] Craige B., Tsao C.C., Diener D.R., Hou Y., Lechtreck K.F., Rosenbaum J.L., Witman G.B. (2010). CEP290 tethers flagellar transition zone microtubules to the membrane and regulates flagellar protein content. J. Cell Biol..

[bib25] Crouse J.A., Lopes V.S., Sanagustin J.T., Keady B.T., Williams D.S., Pazour G.J. (2014). Distinct functions for IFT140 and IFT20 in opsin transport. Cytoskelet. Hob..

[bib26] Deretic D., Huber L.A., Ransom N., Mancini M., Simons K., Papermaster D.S. (1995). rab8 in retinal photoreceptors may participate in rhodopsin transport and in rod outer segment disk morphogenesis. J. Cell Sci..

[bib27] Dry K.L., Manson F.D., Lennon A., Bergen A.A., Van Dorp D.B., Wright A.F. (1999). Identification of a 5' splice site mutation in the RPGR gene in a family with X-linked retinitis pigmentosa (RP3). Hum. Mutat..

[bib28] Dryja T.P., Adams S.M., Grimsby J.L., McGee T.L., Hong D.H., Li T., Andréasson S., Berson E.L. (2001). Null RPGRIP1 alleles in patients with Leber congenital amaurosis. Am. J. Hum. Genet..

[bib29] Eblimit A., Nguyen T.M., Chen Y., Esteve-Rudd J., Zhong H., Letteboer S., Van Reeuwijk J., Simons D.L., Ding Q., Wu K.M., Li Y., Van Beersum S., Moayedi Y., Xu H., Pickard P., Wang K., Gan L., Wu S.M., Williams D.S., Mardon G., Roepman R., Chen R. (2015). Spata7 is a retinal ciliopathy gene critical for correct RPGRIP1 localization and protein trafficking in the retina. Hum. Mol. Genet..

[bib30] Fahim A.T., Bowne S.J., Sullivan L.S., Webb K.D., Williams J.T., Wheaton D.K., Birch D.G., Daiger S.P. (2011). Allelic heterogeneity and genetic modifier loci contribute to clinical variation in males with X-linked retinitis pigmentosa due to RPGR mutations. PLoS One.

[bib31] Gakovic M., Shu X., Kasioulis I., Carpanini S., Moraga I., Wright A.F. (2011). The role of RPGR in cilia formation and actin stability. Hum. Mol. Genet..

[bib32] Gerber S., Perrault I., Hanein S., Barbet F., Ducroq D., Ghazi I., Martin-Coignard D., Leowski C., Homfray T., Dufier J.L., Munnich A., Kaplan J., Rozet J.M. (2001). Complete exon-intron structure of the RPGR-interacting protein (RPGRIP1) gene allows the identification of mutations underlying Leber congenital amaurosis. Eur. J. Hum. Genet..

[bib33] Ghosh A.K., Murga-Zamalloa C.A., Chan L., Hitchcock P.F., Swaroop A., Khanna H. (2010). Human retinopathy-associated ciliary protein retinitis pigmentosa GTPase regulator mediates cilia-dependent vertebrate development. Hum. Mol. Genet..

[bib34] Gonzalez-Cordero A., West E.L., Pearson R.A., Duran Y., Carvalho L.S., Chu C.J., Naeem A., Blackford S.J., Georgiadis A., Lakowski J., Hubank M., Smith A.J., Bainbridge J.W., Sowden J.C., Ali R.R. (2013). Photoreceptor precursors derived from three-dimensional embryonic stem cell cultures integrate and mature within adult degenerate retina. Nat. Biotechnol..

[bib35] Gurdon J. (1962). The developmental capacity of nuclei taken from intestinal epithelium cells of feeding tadpoles. J. Embryol. Exp. Morphol..

[bib36] Hameed A., Abid A., Aziz A., Ismail M., Mehdi S.Q., Khaliq S. (2003). Evidence of RPGRIP1 gene mutations associated with recessive cone-rod dystrophy. J. Med. Genet..

[bib37] Hattula K., Furuhjelm J., Arffman A., Peränen J. (2002). A Rab8-specific GDP/GTP exchange factor is involved in actin remodeling and polarized membrane transport. Mol. Biol. Cell..

[bib38] Hattula K., Furuhjelm J., Tikkanen J., Tanhuanpää K., Laakkonen P., Peränen J. (2006). Characterisation of the Rab8-specific membrane traffic route linked to protrusion formation. J Cell Sci..

[bib39] Hong D.H., Pawlyk B.S., Shang J., Sandberg M.A., Berson E.L., Li T. (2000). A retinitis pigmentosa GTPase regulator (RPGR)-deficient mouse model for X-linked retinitis pigmentosa (RP3). Proc. Natl. Acad. Sci. U. S. A..

[bib40] Hong D.H., Yue G., Adamian M., Li T. (2001). Retinitis pigmentosa GTPase regulator (RPGR)-interacting protein is stably associated with the photoreceptor ciliary axoneme and anchors RPGR to the connecting cilium. J. Biol. Chem..

[bib41] Hong D.H., Pawlyk B., Sokolov M., Strissel K.J., Yang J., Tulloch B., Wright A.F., Arshavsky V.Y., Li T. (2003). RPGR isoforms in photoreceptor connecting cilia and the transitional zone of motile cilia. Invest Ophthalmol. Vis. Sci..

[bib42] Hong D.H., Pawlyk B.S., Adamian M., Li T. (2004). Dominant, gain-of-function mutant produced by truncation of RPGR. Invest Ophthalmol. Vis. Sci..

[bib43] Hong D.H., Pawlyk B.S., Adamian M., Sandberg M.A., Li T. (2005). A single, abbreviated RPGR-ORF15 variant reconstitutes RPGR function in vivo. Invest Ophthalmol. Vis. Sci..

[bib44] Huang W.C., Wright A.F., Roman A.J., Cideciyan A.V., Manson F.D., Gewaily D.Y., Schwartz S.B., Sadigh S., Limberis M.P., Bell P., Wilson J.M., Swaroop A., Jacobson S.G. (2012). RPGR-associated retinal degeneration in human X-linked RP and a murine model. Invest Ophthalmol. Vis. Sci..

[bib45] Iannaccone A., Breuer D.K., Wang X.F., Kuo S.F., Normando E.M., Filippova E., Baldi A., Hiriyanna S., MacDonald C.B., Baldi F., Cosgrove D., Morton C.C., Swaroop A., Jablonski M.M. (2003). Clinical and immunohistochemical evidence for an X linked retinitis pigmentosa syndrome with recurrent infections and hearing loss in association with an RPGR mutation. J. Med. Genet..

[bib46] Jacobson S.G., Cideciyan A.V., Ratnakaram R., Heon E., Schwartz S.B., Roman A.J., Peden M.C., Aleman T.S., Boye S.L., Sumaroka A., Conlon T.J., Calcedo R., Pang J.J., Erger K.E., Olivares M.B., Mullins C.L., Swider M., Kaushal S., Feuer W.J., Iannaccone A., Fishman G.A., Stone E.M., Byrne B.J., Hauswirth W.W. (2012). Gene therapy for leber congenital amaurosis caused by RPE65 mutations: safety and efficacy in 15 children and adults followed up to 3 years. Arch. Ophthalmol..

[bib47] Jackson P.K. (2013). Nek8 couples renal ciliopathies to DNA damage and checkpoint control. Mol. Cell..

[bib48] Kajiwara K., Berson E.L., Dryja T.P. (1994). Digenic retinitis pigmentosa due to mutations at the unlinked peripherin/RDS and ROM1 loci. Science.

[bib49] Keady B.T., Le Y.Z., Pazour G.J. (2011). IFT20 is required for opsin trafficking and photoreceptor outer segment development. Mol. Biol. Cell..

[bib50] Khanna H., Hurd T.W., Lillo C., Shu X., Parapuram S.K., He S., Akimoto M., Wright A.F., Margolis B., Williams D.S., Swaroop A. (2005). RPGR-ORF15, which is mutated in retinitis pigmentosa, associates with SMC1, SMC3, and microtubule transport proteins. J. Biol. Chem..

[bib51] Kim J., Lee J.E., Heynen-Genel S., Suyama E., Ono K., Lee K., Ideker T., Aza-Blanc P., Gleeson J.G. (2010). Functional genomic screen for modulators of ciliogenesis and cilium length. Nature.

[bib52] Kirschner R., Rosenberg T., Schultz-Heienbrok R., Lenzner S., Feil S., Roepman R., Cremers F.P., Ropers H.H., Berger W. (1999). RPGR transcription studies in mouse and human tissues reveal a retina-specific isoform that is disrupted in a patient with X-linked retinitis pigmentosa. Hum. Mol. Genet..

[bib53] Knödler A., Feng S., Zhang J., Zhang X., Das A., Peränen J., Guo W. (2010). Coordination of Rab8 and Rab11 in primary ciliogenesis. Proc. Natl. Acad. Sci. U. S. A..

[bib54] Kremer H., van Wijk E., Märker T., Wolfrum U., Roepman R. (2006). Usher syndrome: molecular links of pathogenesis, proteins and pathways. Hum. Mol. Genet..

[bib55] Lamba D.A., Karl M.O., Ware C.B., Reh T.A. (2006). Efficient generation of retinal progenitor cells from human embryonic stem cells. Proc. Natl. Acad. Sci. U. S. A..

[bib56] Li T. (2014). Leber congenital amaurosis caused by mutations in RPGRIP1. Cold Spring Harb. Perspect. Med..

[bib57] Linari M., Ueffing M., Manson F., Wright A., Meitinger T., Becker J. (1999). The retinitis pigmentosa GTPase regulator, RPGR, interacts with the delta subunit of rod cyclic GMP phosphodiesterase. Proc. Natl. Acad. Sci. U. S. A..

[bib58] Lu X., Guruju M., Oswald J., Ferreira P.A. (2005). Limited proteolysis differentially modulates the stability and subcellular localization of domains of RPGRIP1 that are distinctly affected by mutations in Leber's congenital amaurosis. Hum. Mol. Genet..

[bib59] MacLaren R.E., Pearson R.A., MacNeil A., Douglas R.H., Salt T.E., Akimoto M., Swaroop A., Sowden J.C., Ali R.R. (2006). Retinal repair by transplantation of photoreceptor precursors. Nature.

[bib60] Maerker T., van Wijk E., Overlack N., Kersten F.F., McGee J., Goldmann T., Sehn E., Roepman R., Walsh E.J., Kremer H., Wolfrum U. (2008). A novel Usher protein network at the periciliary reloading point between molecular transport machineries in vertebrate photoreceptor cells. Hum. Mol. Genet..

[bib61] Maguire A.M., Simonelli F., Pierce E.A., Pugh E.N., Mingozzi F., Bennicelli J., Banfi S., Marshall K.A., Testa F., Surace E.M., Rossi S., Lyubarsky A., Arruda V.R., Konkle B., Stone E., Sun J., Jacobs J., Dell'Osso L., Hertle R., Ma J.X., Redmond T.M., Zhu X., Hauck B., Zelenaia O., Shindler K.S., Maguire M.G., Wright J.F., Volpe N.J., McDonnell J.W., Auricchio A., High K.A., Bennett J. (2008). Safety and efficacy of gene transfer for Leber's congenital amaurosis. New. Engl. J. Med..

[bib62] Mavlyutov T.A., Zhao H., Ferreira P.A. (2002). Species-specific subcellular localization of RPGR and RPGRIP isoforms: implications for the phenotypic variability of congenital retinopathies among species. Hum. Mol. Genet..

[bib63] Mburu P., Kikkawa Y., Townsend S., Romero R., Yonekawa H., Brown S.D. (2006). Whirlin complexes with p55 at the stereocilia tip during hair cell development. Proc. Natl. Acad. Sci. U. S. A..

[bib64] Meindl A., Dry K., Herrmann K., Manson F., Ciccodicola A., Edgar A., Carvalho M.R., Achatz H., Hellebrand H., Lennon A., Migliaccio C., Porter K., Zrenner E., Bird A., Jay M., Lorenz B., Wittwer B., D'Urso M., Meitinger T., Wright A.F. (1996). A gene (RPGR) with homology to the RCC1 guanine nucleotide exchange factor is mutated in X-linked retinitis pigmentosa (RP3). Nat. Genet..

[bib65] Mellough C.B., Sernagor E., Moreno-Gimeno I., Steel D.H., Lako M. (2012). Efficient stage-specific differentiation of human pluripotent stem cells toward retinal photoreceptor cells. Stem Cells.

[bib66] Moritz O.L.1, Tam B.M., Hurd L.L., Peränen J., Deretic D., Papermaster D.S. (2001). Mutant rab8 impairs docking and fusion of rhodopsin-bearing post-golgi membranes and causes cell death of transgenic Xenopus rods. Mol. Biol. Cell..

[bib67] Murga-Zamalloa C.A., Desai N.J., Hildebrandt F., Khanna H. (2010). Interaction of ciliary disease protein retinitis pigmentosa GTPase regulator with nephronophthisis-associated proteins in mammalian retinas. Mol. Vis..

[bib68] Murga-Zamalloa C.A., Atkins S.J., Peranen J., Swaroop A., Khanna H. (2010). Interaction of retinitis pigmentosa GTPase regulator (RPGR) with RAB8A GTPase: implications for cilia dysfunction and photoreceptor degeneration. Hum. Mol. Genet..

[bib69] Nakano T., Ando S., Takata N., Kawada M., Muguruma K., Sekiguchi K., Saito K., Yonemura S., Eiraku M., Sasai Y. (2012). Self-formation of optic cups and storable stratified neural retina from human ESCs. Cell Stem Cell.

[bib70] Neidhardt J., Glaus E., Barthelmes D., Zeitz C., Fleischhauer J., Berger W. (2007). Identification and characterization of a novel RPGR isoform in human retina. Hum. Mutat..

[bib71] Osakada F., Ikeda H., Mandai M., Wataya T., Watanabe K., Yoshimura N., Akaike A., Sasai Y., Takahashi M. (2008). Toward the generation of rod and cone photoreceptors from mouse, monkey and human embryonic stem cells. Nat. Biotechnol..

[bib72] Otto E.A., Loeys B., Khanna H., Hellemans J., Sudbrak R., Fan S., Muerb U., O'Toole J.F., Helou J., Attanasio M., Utsch B., Sayer J.A., Lillo C., Jimeno D., Coucke P., De Paepe A., Reinhardt R., Klages S., Tsuda M., Kawakami I., Kusakabe T., Omran H., Imm A., Tippens M., Raymond P.A., Hill J., Beales P., He S., Kispert A., Margolis B., Williams D.S., Swaroop A., Hildebrandt F. (2005). Nephrocystin-5, a ciliary IQ domain protein, is mutated in senior-loken syndrome and interacts with RPGR and calmodulin. Nat. Genet..

[bib74] Patil H., Guruju M.R., Cho K.I., Yi H., Orry A., Kim H., Ferreira P.A. (2012). Structural and functional plasticity of subcellular tethering, targeting and processing of RPGRIP1 by RPGR isoforms. Biol. Open.

[bib75] Patil H., Tserentsoodol N., Saha A., Hao Y., Webb M., Ferreira P.A. (2012). Selective loss of RPGRIP1-dependent ciliary targeting of NPHP4, RPGR and SDCCAG8 underlies the degeneration of photoreceptor neurons. Cell Death Dis..

[bib73] Pearson R.A., Barber A.C., Rizzi M., Hippert C., Xue T., West E.L., Duran Y., Smith A.J., Chuang J.Z., Azam S.A., Luhmann U.F., Benucci A., Sung C.H., Bainbridge J.W., Carandini M., Yau K.W., Sowden J.C., Ali R.R. (2012). Restoration of vision after transplantation of photoreceptors. Nature.

[bib76] Peters K.R., Palade G.E., Schneider B.G., Papermaster D.S. (1983). Fine structure of a periciliary ridge complex of frog retinal rod cells revealed by ultrahigh resolution scanning electron microscopy. J. Cell Biol..

[bib77] Rachel R.A., Li T., Swaroop A. (2012). Photoreceptor sensory cilia and ciliopathies: focus on CEP290, RPGR and their interacting proteins. Cilia.

[bib78] Reiter J.F., Blacque O.E., Leroux M.R. (2012). The base of the cilium: roles for transition fibres and the transition zone in ciliary formation, maintenance and compartmentalization. EMBO Rep..

[bib79] Remans K., Bürger M., Vetter I.R., Wittinghofer A. (2014). C2 domains as protein-protein interaction modules in the ciliary transition zone. Cell Rep..

[bib80] Renault L., Nassar N., Vetter I., Becker J., Klebe C., Roth M., Wittinghofer A. (1998). The 1.7 A crystal structure of the regulator of chromosome condensation (RCC1) reveals a seven-bladed propeller. Nature.

[bib123] Roepman R., van Duijnhoven G., Rosenberg T., Pinckers A.J., Bleeker-Wagemakers L.M., Bergen A.A., Post J., Beck A., Reinhardt R., Ropers H.H., Cremers F.P., Berger W. (1996). Positional cloning of the gene for X-linked retinitis pigmentosa 3: homology with the guanine-nucleotide-exchange factor RCC1.. Hum. Mol. Genet..

[bib81] Roepman R., Bernoud-Hubac N., Schick D.E., Maugeri A., Berger W., Ropers H.H., Cremers F.P., Ferreira P.A. (2000). The retinitis pigmentosa GTPase regulator (RPGR) interacts with novel transport-like proteins in the outer segments of rod photoreceptors. Hum. Mol. Genet..

[bib82] Sahel J.A., Marazova K., Audo I. (2014). Clinical characteristics and current therapies for inherited retinal degenerations. Cold Spring Harb. Perspect. Med..

[bib83] Sang L., Miller J.J., Corbit K.C., Giles R.H., Brauer M.J., Otto E.A., Baye L.M., Wen X., Scales S.J., Kwong M., Huntzicker E.G., Sfakianos M.K., Sandoval W., Bazan J.F., Kulkarni P., Garcia-Gonzalo F.R., Seol A.D., O'Toole J.F., Held S., Reutter H.M., Lane W.S., Rafiq M.A., Noor A., Ansar M., Devi A.R., Sheffield V.C., Slusarski D.C., Vincent J.B., Doherty D.A., Hildebrandt F., Reiter J.F., Jackson P.K. (2011). Mapping the NPHP-JBTS-MKS protein network reveals ciliopathy disease genes and pathways. Cell.

[bib84] Sayer J.A., Otto E.A., O'Toole J.F., Nurnberg G., Kennedy M.A., Becker C., Hennies H.C., Helou J., Attanasio M., Fausett B.V., Utsch B., Khanna H., Liu Y., Drummond I., Kawakami I., Kusakabe T., Tsuda M., Ma L., Lee H., Larson R.G., Allen S.J., Wilkinson C.J., Nigg E.A., Shou C., Lillo C., Williams D.S., Hoppe B., Kemper M.J., Neuhaus T., Parisi M.A., Glass I.A., Petry M., Kispert A., Gloy J., Ganner A., Walz G., Zhu X., Goldman D., Nurnberg P., Swaroop A., Leroux M.R., Hildebrandt F. (2006). The centrosomal protein nephrocystin-6 is mutated in Joubert syndrome and activates transcription factor ATF4. Nat. Genet..

[bib86] Schmid F., Glaus E., Cremers F.P., Kloeckener-Gruissem B., Berger W., Neidhardt J. (2010). Mutation- and tissue-specific alterations of RPGR transcripts. Invest Ophthalmol. Vis. Sci..

[bib87] Schwartz S.D., Regillo C.D., Lam B.L., Eliott D., Rosenfeld P.J., Gregori N.Z., Hubschman J.P., Davis J.L., Heilwell G., Spirn M., Maguire J., Gay R., Bateman J., Ostrick R.M., Morris D., Vincent M., Anglade E., Del Priore L.V., Lanza R. (2015). Human embryonic stem cell-derived retinal pigment epithelium in patients with age-related macular degeneration and Stargardt's macular dystrophy: follow-up of two open-label phase 1/2 studies. Lancet.

[bib85] Sharon D., Sandberg M.A., Rabe V.W., Stillberger M., Dryja T.P., Berson E.L. (2003). RP2 and RPGR mutations and clinical correlations in patients with X-linked retinitis pigmentosa. Am. J. Hum. Genet..

[bib88] Shu X., Fry A.M., Tulloch B., Manson F.D., Crabb J.W., Khanna H., Faragher A.J., Lennon A., He S., Trojan P., Giessl A., Wolfrum U., Vervoort R., Swaroop A., Wright A.F. (2005). RPGR ORF15 isoform co-localizes with RPGRIP1 at centrioles and basal bodies and interacts with nucleophosmin. Hum. Mol. Genet..

[bib89] Shu X., Zeng Z., Eckmiller M.S., Gautier P., Vlachantoni D., Manson F.D., Tulloch B., Sharpe C., Gorecki D.C., Wright A.F. (2006). Developmental and tissue expression of Xenopus laevis RPGR. Invest Ophthalmol. Vis. Sci..

[bib90] Shu X., Zeng Z., Gautier P., Lennon A., Gakovic M., Patton E.E., Wright A.F. (2010). Zebrafish Rpgr is required for normal retinal development and plays a role in dynein-based retrograde transport processes. Hum. Mol. Genet..

[bib91] Shu X., Simpson J.R., Hart A.W., Zeng Z., Patnaik S.R., Gautier P., Murdoch E., Tulloch B., Wright A.F. (2012). Functional characterization of the human RPGR proximal promoter. Invest Ophthalmol. Vis. Sci..

[bib124] Shu X., Black G.C., Rice J.M., Hart-Holden N., Jones A., O'Grady A., Ramsden S., Wright A.F. (2007). RPGR mutation analysis and disease: an update. Hum. Mutat..

[bib92] Singh M.S., Charbel Issa P., Butler R., Martin C., Lipinski D.M., Sekaran S., Barnard A.R., MacLaren R.E. (2013). Reversal of end-stage retinal degeneration and restoration of visual function by photoreceptor transplantation. Proc. Natl. Acad. Sci. U. S. A..

[bib93] Stenson P.D., Mort M., Ball E.V., Shaw K., Phillips A., Cooper D.N. (2014). The human gene mutation database: building a comprehensive mutation repository for clinical and molecular genetics, diagnostic testing and personalized genomic medicine. Hum. Genet..

[bib94] Sung C.H., Leroux M.R. (2013). The roles of evolutionarily conserved functional modules in cilia-related trafficking. Nat. Cell Biol..

[bib95] Szklarczyk D., Franceschini A., Wyder S., Forslund K., Heller D., Huerta-Cepas J., Simonovic M., Roth A., Santos A., Tsafou K., Kuhn M., Bork P., Jensen L., von Mering C. (2015). STRING v10: protein-protein interaction networks, integrated over the tree of life. Nucleic Acids Res..

[bib96] Takahashi K., Tanabe K., Ohnuki M., Narita M., Ichisaka T., Tomoda K., Yamanaka S. (2007). Induction of pluripotent stem cells from adult human fibroblasts by defined factors. Cell.

[bib97] Thomas S., Wright K.J., Le Corre S., Micalizzi A., Romani M., Abhyankar A., Saada J., Perrault I., Amiel J., Litzler J., Filhol E., Elkhartoufi N., Kwong M., Casanova J.L., Boddaert N., Baehr W., Lyonnet S., Munnich A., Burglen L., Chassaing N., Encha-Ravazi F., Vekemans M., Gleeson J.G., Valente E.M., Jackson P.K., Drummond I.A., Saunier S., Attié-Bitach T. (2014). A homozygous PDE6D mutation in Joubert syndrome impairs targeting of farnesylated INPP5E protein to the primary cilium. Hum. Mutat..

[bib98] Thompson D.A.1, Khan N.W., Othman M.I., Chang B., Jia L., Grahek G., Wu Z., Hiriyanna S., Nellissery J., Li T., Khanna H., Colosi P., Swaroop A., Heckenlively J.R. (2012). Rd9 is a naturally occurring mouse model of a common form of retinitis pigmentosa caused by mutations in RPGR-ORF15. PLoS One.

[bib99] Valdés-Sánchez L., De la Cerda B., Diaz-Corrales F.J., Massalini S., Chakarova C.F., Wright A.F., Bhattacharya S.S. (2013). ATR localizes to the photoreceptor connecting cilium and deficiency leads to severe photoreceptor degeneration in mice. Hum. Mol. Genet..

[bib100] van Wijk E., van der Zwaag B., Peters T., Zimmermann U., Te Brinke H., Kersten F.F., Märker T., Aller E., Hoefsloot L.H., Cremers C.W., Cremers F.P., Wolfrum U., Knipper M., Roepman R., Kremer H. (2006). The DFNB31 gene product whirlin connects to the Usher protein network in the cochlea and retina by direct association with USH2A and VLGR1. Hum. Mol. Genet..

[bib101] Vaughan D.K., Fisher S.K. (1989). Cytochalasin D disrupts outer segment disc morphogenesis in situ in rabbit retina. Invest Ophthalmol. Vis. Sci..

[bib102] Vervoort R., Lennon A., Bird A.C., Tulloch B., Axton R., Miano M.G., Meindl A., Meitinger T., Ciccodicola A., Wright A.F. (2000). Mutational hot spot within a new RPGR exon in X-linked retinitis pigmentosa. Nat. Genet..

[bib103] Walia S., Fishman G.A., Swaroop A., Branham K.E., Lindeman M., Othman M., Weleber R.G. (2008). Discordant phenotypes in fraternal twins having an identical mutation in exon ORF15 of the RPGR gene. Arch. Ophthalmol..

[bib104] Wang L., Zou J., Shen Z., Song E., Yang J. (2012). Whirlin interacts with espin and modulates its actin-regulatory function: an insight into the mechanism of Usher syndrome type II. Hum. Mol. Genet..

[bib105] Warde-Farley D., Donaldson S.L., Comes O., Zuberi K., Badrawi R., Chao P., Franz M., Grouios C., Kazi F., Lopes C.T., Maitland A., Mostafavi S., Montojo J., Shao Q., Wright G., Bader G.D., Morris Q. (2010). The GeneMANIA prediction server: biological network integration for gene prioritization and predicting gene function. Nucleic Acids Res..

[bib106] Wätzlich D., Vetter I., Gotthardt K., Miertzschke M., Chen Y.X., Wittinghofer A., Ismail S. (2013). The interplay between RPGR, PDEδ and Arl2/3 regulate the ciliary targeting of farnesylated cargo. EMBO Rep..

[bib108] Williams D.S., Linberg K.A., Vaughan D.K., Fariss R.N., Fisher S.K. (1988). Disruption of microfilament organization and deregulation of disk membrane morphogenesis by cytochalasin D in rod and cone photoreceptors. J. Comp. Neurol..

[bib109] Wilmut I., Schnieke A.E., McWhir J., Kind A.J., Campbell K.H. (1997). Viable offspring derived from fetal and adult mammalian cells. Nature.

[bib107] Won J., Gifford E., Smith R.S., Yi H., Ferreira P.A., Hicks W.L., Li T., Naggert J.K., Nishina P.M. (2009). RPGRIP1 is essential for normal rod photoreceptor outer segment elaboration and morphogenesis. Hum. Mol. Genet..

[bib110] Wright A.F., Shu X. (2007). Focus on molecules: RPGR. Exp. Eye Res..

[bib111] Wright A.F., Chakarova C.F., Abd El-Aziz M.M., Bhattacharya S.S. (2010). Photoreceptor degeneration: genetic and mechanistic dissection of a complex trait. Nat. Rev. Genet..

[bib112] Wright R.N., Hong D., Perkins (2011). Misexpression of the constitutive Rpgrex1-19 variant leads to severe photoreceptor degeneration. Invest Ophthalmol. Vis. Sci..

[bib113] Wright R.N., Hong D., Perkins B. (2012). RpgrORF15 connects to the Usher protein network through direct interactions with multiple whirlin isoforms. Invest Ophthalmol. Vis. Sci..

[bib114] Yan D., Swain P.K., Breuer D., Tucker R.M., Wu W., Fujita R., Rehemtulla A., Burke D., Swaroop A. (1998). Biochemical characterization and subcellular localization of the mouse retinitis pigmentosa GTPase regulator (mRpgr). J. Biol. Chem..

[bib115] Yang J., Liu X., Zhao Y., Adamian M., Pawlyk B., Sun X., McMillan D.R., Liberman M.C., Li T. (2010). Ablation of whirlin long isoform disrupts the USH2 protein complex and causes vision and hearing loss. PLoS Genet..

[bib116] Zahid S., Khan N., Branham K., Othman M., Karoukis A.J., Sharma N., Moncrief A., Mahmood M.N., Sieving P.A., Swaroop A., Heckenlively J.R., Jayasundera T. (2013). Phenotypic conservation in patients with X-linked retinitis pigmentosa caused by RPGR mutations. JAMA Ophthalmol..

[bib117] Zeiss C.J., Acland G.M., Aguirre G.D. (1999). Retinal pathology of canine X-linked progressive retinal atrophy, the locus homologue of RP3. Invest Ophthalmol. Vis. Sci..

[bib118] Zhang Q., Acland G.M., Wu W.X., Johnson J.L., Pearce-Kelling S., Tulloch B., Vervoort R., Wright A.F., Aguirre G.D. (2002). Different RPGR exon ORF15 mutations in canids provide insights into photoreceptor cell degeneration. Hum. Mol. Genet..

[bib119] Zhang H., Li S., Doan T., Rieke F., Detwiler P.B., Frederick J.M., Baehr W. (2007). Deletion of PrBP/delta impedes transport of GRK1 and PDE6 catalytic subunits to photoreceptor outer segments. Proc. Natl. Acad. Sci. U. S. A..

[bib120] Zhao Y., Hong D.H., Pawlyk B., Yue G., Adamian M., Grynberg M., Godzik A., Li T. (2003). The retinitis pigmentosa GTPase regulator (RPGR)-interacting protein: subserving RPGR function and participating in disk morphogenesis. Proc. Natl. Acad. Sci. U. S. A..

[bib121] Zhong X., Gutierrez C., Xue T., Hampton C., Vergara M.N., Cao L.H., Peters A., Park T.S., Zambidis E.T., Meyer J.S., Gamm D.M., Yau K.W., Canto-Soler M.V. (2014). Generation of three-dimensional retinal tissue with functional photoreceptors from human iPSCs. Nat. Commun..

[bib122] Zito I., Downes S.M., Patel R.J., Cheetham M.E., Ebenezer N.D., Jenkins S.A., Bhattacharya S.S., Webster A.R., Holder G.E., Bird A.C., Bamiou D.E., Hardcastle A.J. (2003). RPGR mutation associated with retinitis pigmentosa, impaired hearing, and sinorespiratory infections. J. Med. Genet..

